# Supplementary figures and images for: The RNA-binding protein HuR modulates the expression of the disease-linked CCL2 rs1024611G-rs13900T haplotype
Source: eLife. 2026 Jan 14;13:RP93108. doi: 10.7554/eLife.93108 (PMC12803514; doi:10.7554/eLife.93108)

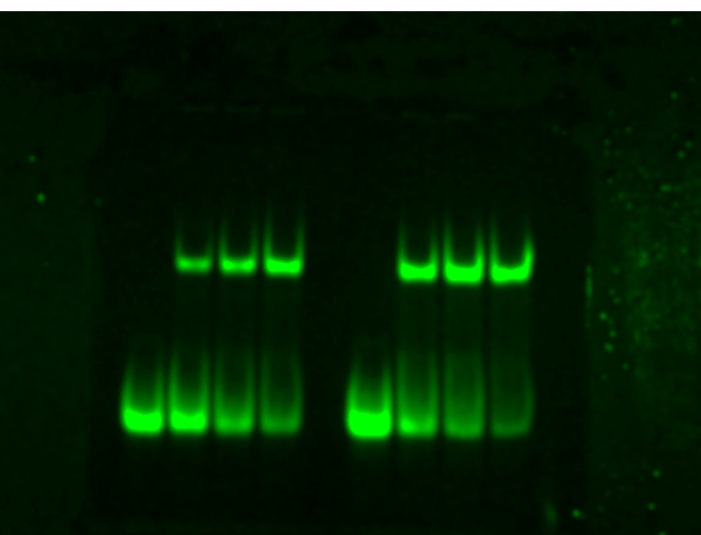

Supplement: Figure 4—source data 2. [file elife-93108-fig4-data2.zip › REMSA_purified recombinant HuR.tif]

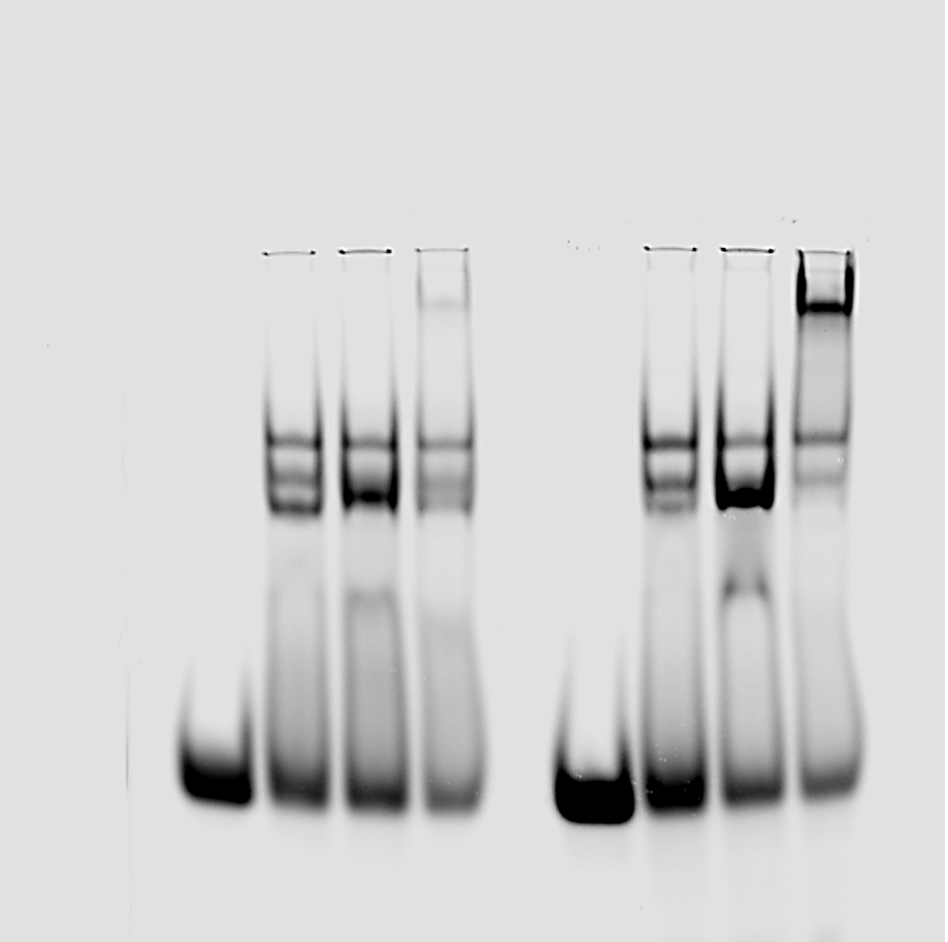

Supplement: Figure 4—source data 2. [file elife-93108-fig4-data2.zip › REMSA_ whole cell extrcat.tif]

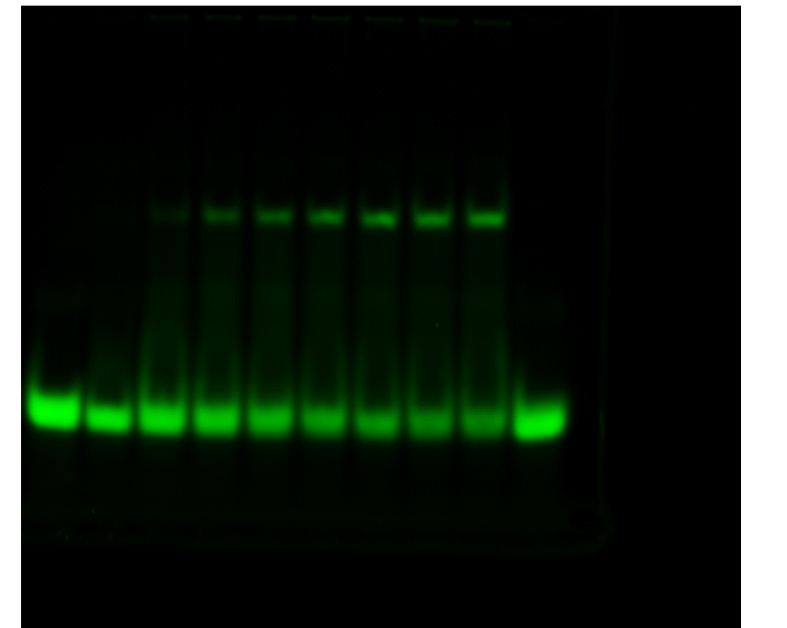

Supplement: Figure 4—figure supplement 1—source data 2. [file elife-93108-fig4-figsupp1-data2.zip › REMSA_purified recombinant HuR_rs13900C.tif]

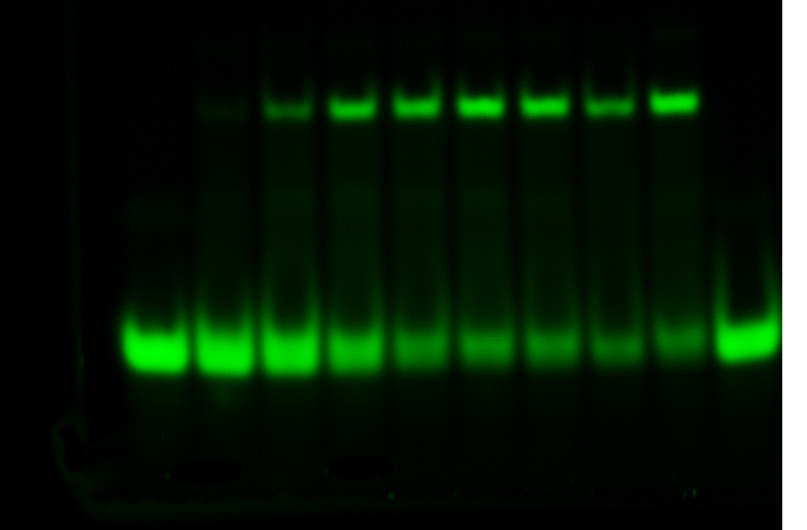

Supplement: Figure 4—figure supplement 1—source data 2. [file elife-93108-fig4-figsupp1-data2.zip › REMSA_purified recombinant HuR_rs13900T.tif]

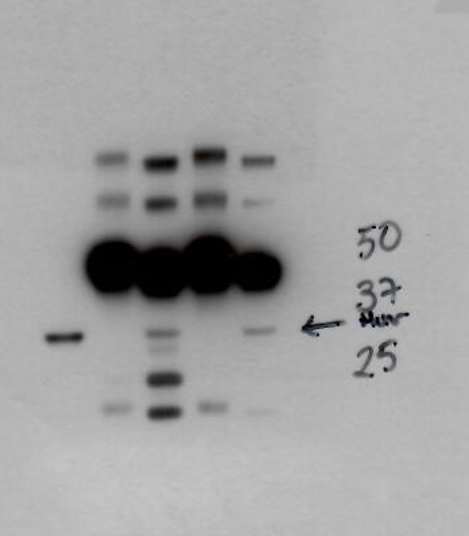

Supplement: Figure 5—source data 2. [file elife-93108-fig5-data2.zip › RIP_HuR enrichment Donor 3-4.tif]

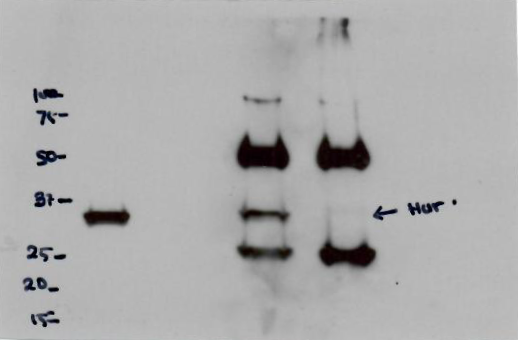

Supplement: Figure 5—source data 2. [file elife-93108-fig5-data2.zip › RIP assay_Quality Check.tiff]

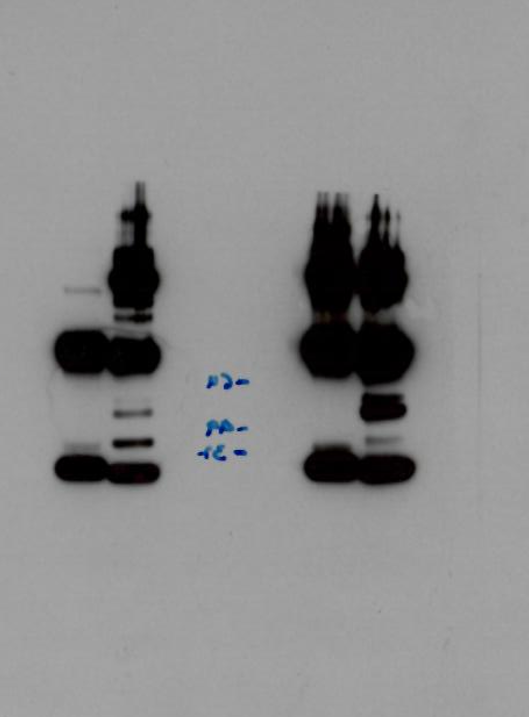

Supplement: Figure 5—source data 2. [file elife-93108-fig5-data2.zip › RIP_HuR enrichment Donor 1-2.tif]

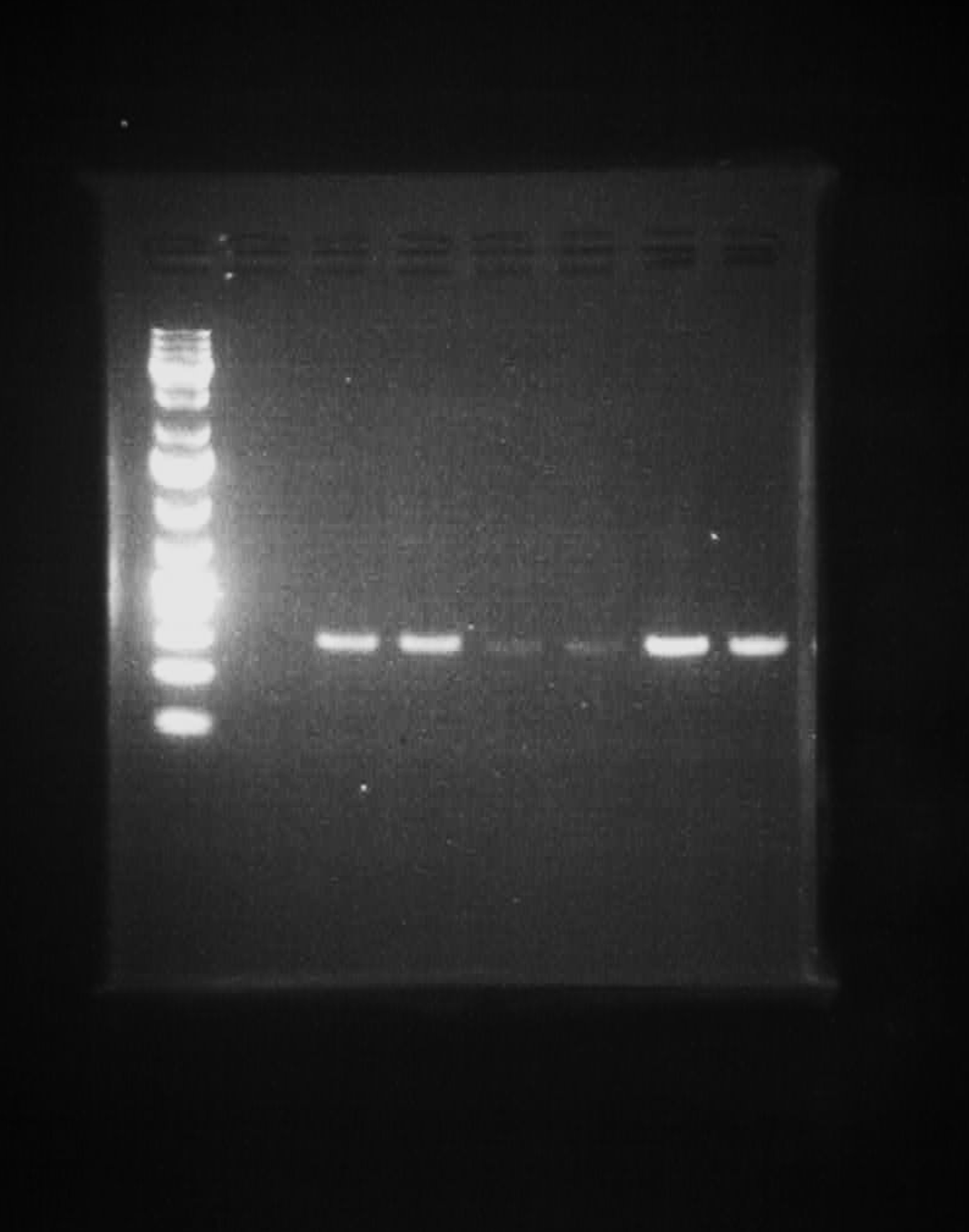

Supplement: Figure 5—source data 4. [file elife-93108-fig5-data4.zip › CCL2 -3'UTR identification.tif]

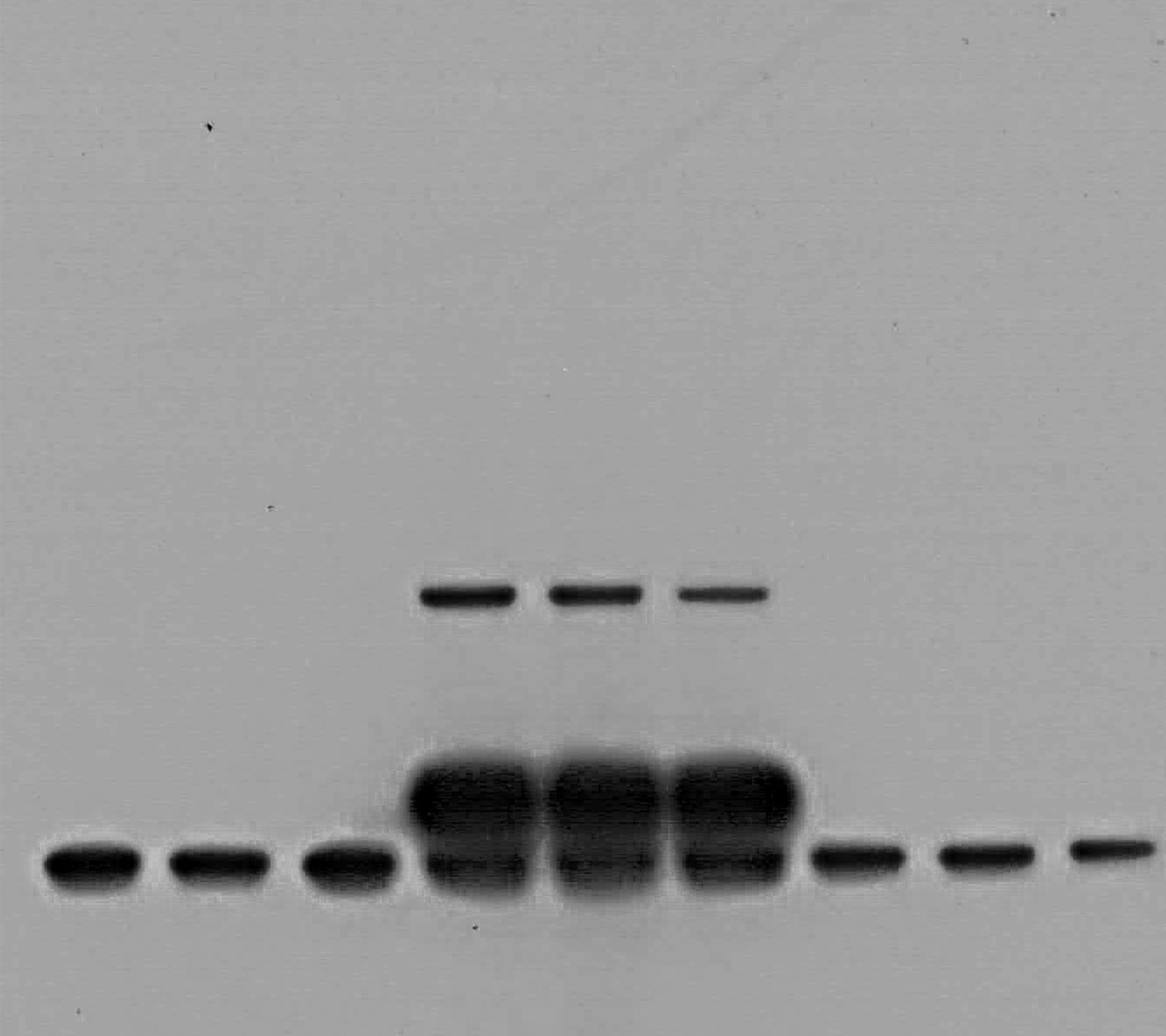

Supplement: Figure 6—figure supplement 1—source data 2. [file elife-93108-fig6-figsupp1-data2.zip › Blot 1.png]

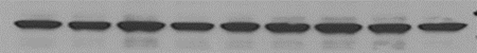

Supplement: Figure 6—figure supplement 1—source data 2. [file elife-93108-fig6-figsupp1-data2.zip › actin.png]
