# Supplementary material for: The RNA-binding protein HuR modulates the expression of the disease-linked CCL2 rs1024611G-rs13900T haplotype
Source: eLife. 2026 Jan 14;13:RP93108. doi: 10.7554/eLife.93108 (PMC12803514; doi:10.7554/eLife.93108)
Supplement: Figure 4—figure supplement 1—source data 1. [file elife-93108-fig4-figsupp1-data1.zip › Figure 4 -figure supplement 1-source data 1.pdf]

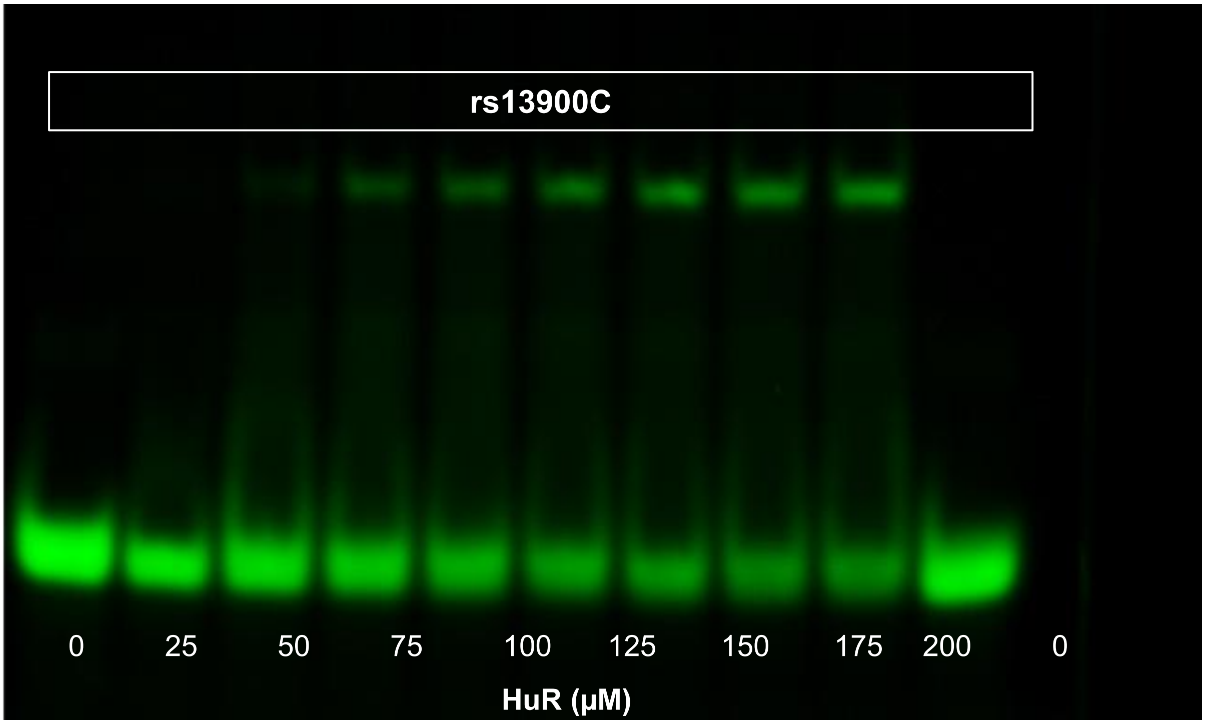

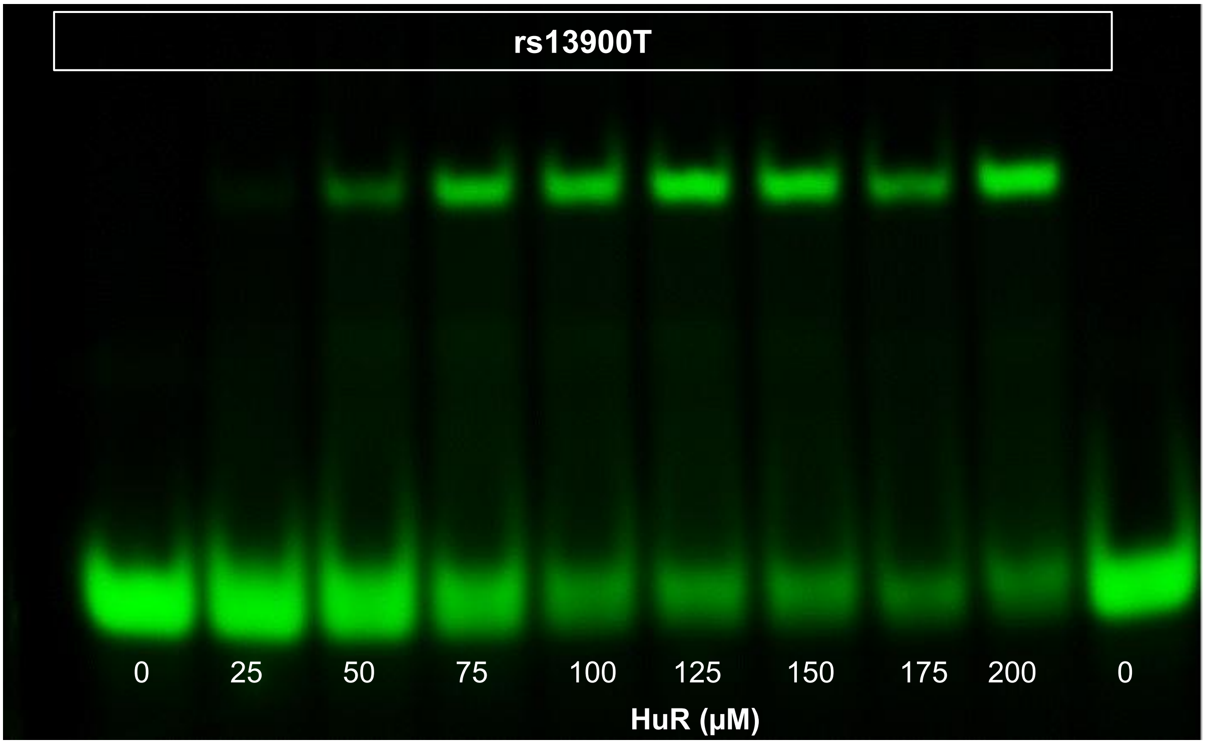

Figure 4 – figure supplement 1-source data 1. Original uncropped membranes corresponding to Figure 4 –figure supplement 1. REMSA using labeled oligoribonucleotides containing either rs13900T or rs13900C allele incubated with purified recombinant HuR at the indicated concentrations.
