# Supplementary material for: The RNA-binding protein HuR modulates the expression of the disease-linked CCL2 rs1024611G-rs13900T haplotype
Source: eLife. 2026 Jan 14;13:RP93108. doi: 10.7554/eLife.93108 (PMC12803514; doi:10.7554/eLife.93108)
Supplement: Figure 5—source data 1. [file elife-93108-fig5-data1.zip › Figure 5 –source data 1.pdf]

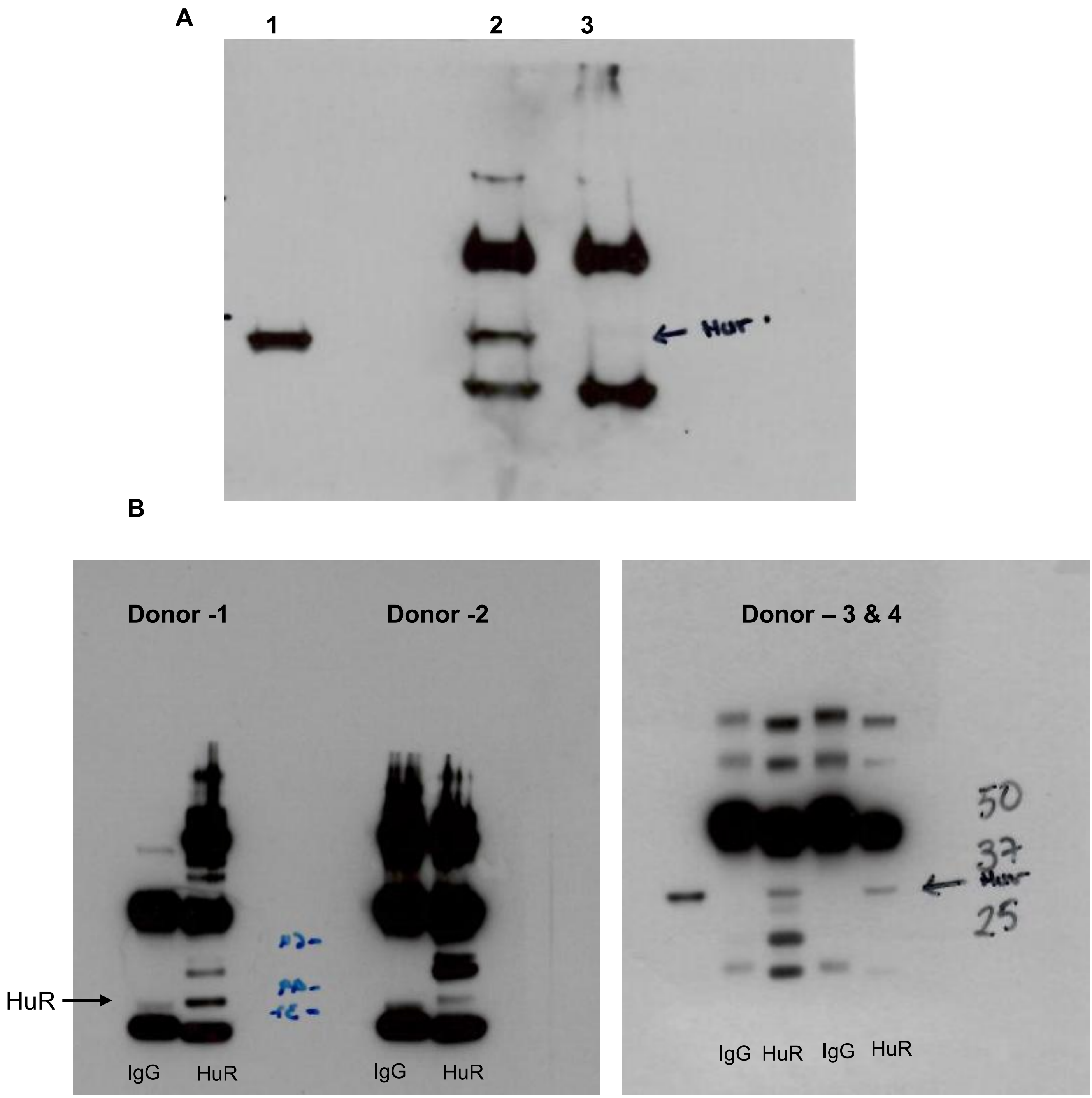

Figure 5 –source data 1. Original uncropped membranes showing HuR enrichment in immunoprecipitated material (supporting data for Figure 5A).

(A) Quality check for RIP assay assessed by Western blot analysis of HuR expression levels by western blotting. Lane 1: Input sample (precleared cell lysate); Lane 2: post-IP beads of anti-HuR antibody; Lane 3: post-IP beads of Normal Rabbit IgG.

(B) Enrichment of HuR-containing RNP complexes following RIP in four heterozygous donors. Western blotting showing the relative enrichment of HuR in α-HuR immunoprecipitated fractions compared to IgG control.
