# Supplementary material for: The RNA-binding protein HuR modulates the expression of the disease-linked CCL2 rs1024611G-rs13900T haplotype
Source: eLife. 2026 Jan 14;13:RP93108. doi: 10.7554/eLife.93108 (PMC12803514; doi:10.7554/eLife.93108)
Supplement: Figure 5—source data 3. [file elife-93108-fig5-data3.zip › Figure 5 –source data 3.pdf]

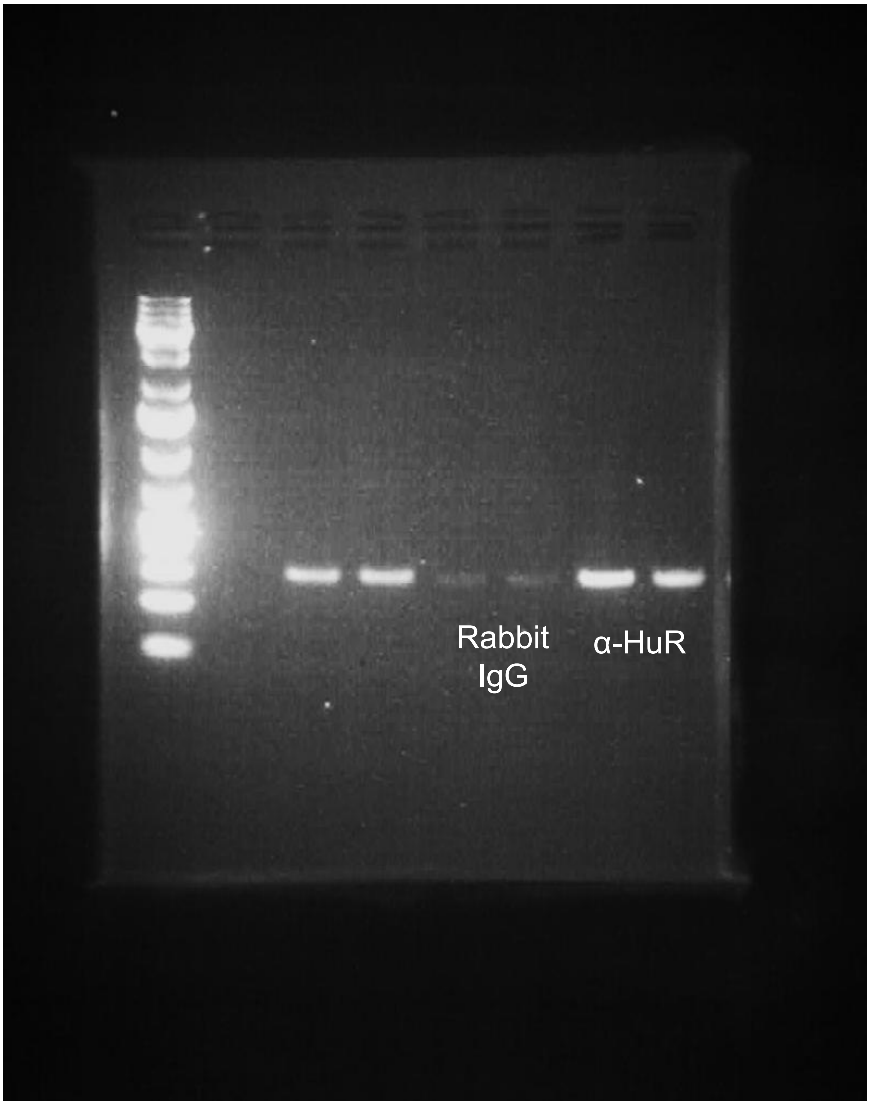

Figure 5 –source data 3. Original uncropped gel showing identification of target RNA (CCL2-3'UTR) from cellular RNP complex. (Figure 5B). The association of HuR with 3'UTR of CCL2 was tested by RIP assay, followed by detection of the target transcript of interest by RT-PCR of RIP materials. PCR products were visualized by electrophoresis in STBR safe stained 2% agarose gel.
