# Supplementary material for: The RNA-binding protein HuR modulates the expression of the disease-linked CCL2 rs1024611G-rs13900T haplotype
Source: eLife. 2026 Jan 14;13:RP93108. doi: 10.7554/eLife.93108 (PMC12803514; doi:10.7554/eLife.93108)
Supplement: Figure 6—figure supplement 1—source data 1. [file elife-93108-fig6-figsupp1-data1.zip › Figure 6-Figure supplement 1 -source data 1.pdf]

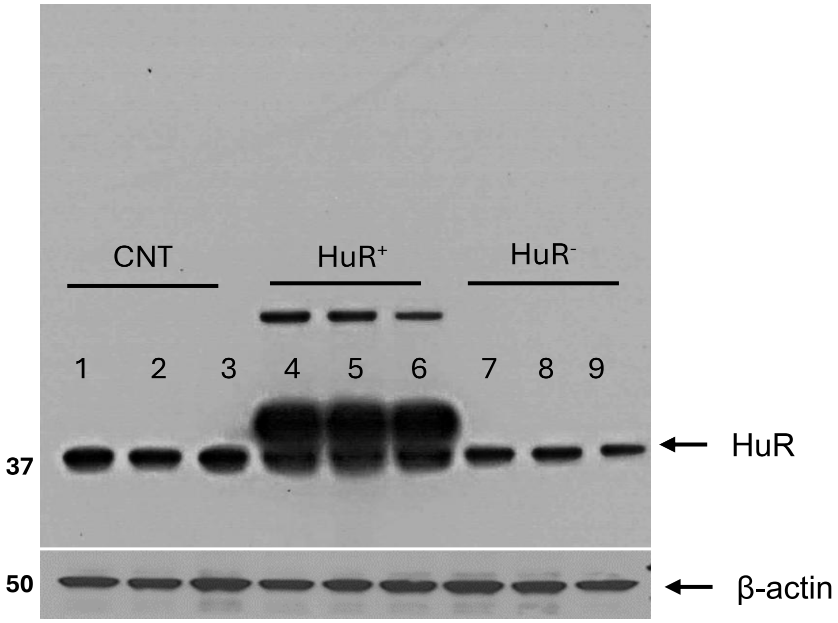

Figure 6 – figure supplement 1 –source data 1. Original uncropped Western blot showing HuR protein levels in HEK 293 cells transfected with either pCMV6-HuR (HuR^{+}) or HuR-targeting siRNA (HuR^{-}).
