# Supplementary material for: The RNA-binding protein HuR modulates the expression of the disease-linked CCL2 rs1024611G-rs13900T haplotype
Source: eLife. 2026 Jan 14;13:RP93108. doi: 10.7554/eLife.93108 (PMC12803514; doi:10.7554/eLife.93108)
Supplement: Supplementary file 1. — MAF, minor allele frequency. [file elife-93108-supp1.docx]

|  | **rs13900** |
| --- | --- |
|  | **(n=42)** |
| Reference allele [CC] | 18 (42.8%) |
| Heterozygous [CT] | 16 (38.09%) |
| Alternative allele [TT] | 8 (19.04%) |
| MAF | 0.38 |
| Heterozygosity | 0.47 |
| No of alleles | 84 |
| Hardy Weinberg *P* | 0.459 |

**Supplementary File 1**. Allele carriages and allele frequencies of rs13900 in healthy volunteers, MAF, minor allele frequency.
