# Supplementary material for: The RNA-binding protein HuR modulates the expression of the disease-linked CCL2 rs1024611G-rs13900T haplotype
Source: eLife. 2026 Jan 14;13:RP93108. doi: 10.7554/eLife.93108 (PMC12803514; doi:10.7554/eLife.93108)
Supplement: Supplementary file 2. — A summary of the single nucleotide polymorphisms (SNPs) located within HuR-binding regions is shown, including SNP ID (rs#), genomic coordinates, strand, binding score, conservation scores (PhastCons, PhyloP), dataset accession numbers, alleles, and SNP position within peak region. Data were generated using the Genomic Variants Module in the POSTAR3 platform. The GSE accession #s correspond to those reported in the Gene Expression Omnibus (GEO). [file elife-93108-supp2.docx]

| **RBP** | **Tissue type** | **rs#** | **Position** | **Strand** | **CLIP-seq technology and**  **peak calling method** | **Score** | **PhastCons score** | **PhyloP score** | **Data accession** | **Ref strand** | **Alt strand** | **SNP position** |
| --- | --- | --- | --- | --- | --- | --- | --- | --- | --- | --- | --- | --- |
| ELAVL1 | HeLa | rs13900 | chr17:34256881-  34256901 | + | PAR-  CLIP,Piranha_0.01 | 11 | 0.024 | 0.227 | GSE29943,GSM741175 | C | T | chr17:34256891-  34256892 |
| ELAVL1 | HeLa | rs181021073 | chr17:34256857-  34256876 | + | PAR-  CLIP,PARalyzer | 0.647 | 0.002 | 0.14 | GSE29943,GSM741173 | T | G | chr17:34256868-  34256869 |
| ELAVL1 | HeLa | rs181021073 | chr17:34256857-  34256876 | + | PAR-  CLIP,PARalyzer | 0.653 | 0.002 | 0.14 | GSE29943,GSM741174 | T | G | chr17:34256868-  34256869 |
| ELAVL1 | HeLa | NA | chr17:34257108-  34257130 | + | PAR-  CLIP,PARalyzer | 0.623 | 0.001 | -0.256 | GSE29943,GSM741173 | AT | A | chr17:34257109-  34257110 |
| ELAVL1 | HeLa | NA | chr17:34257101-  34257121 | + | PAR-  CLIP,Piranha_0.01 | 9 | 0 | -0.39 | GSE29943,GSM741173 | AT | A | chr17:34257109-  34257110 |
| ELAVL1 | HeLa | NA | chr17:34257101-  34257121 | + | PAR-  CLIP,Piranha_0.01 | 6 | 0 | -0.39 | GSE29943,GSM741175 | AT | A | chr17:34257109-  34257110 |

**Supplementary File 2.** Crosslinking immunoprecipitation (CLIP) analysis of HuR binding sites on the 3’untranslated region (3’UTR) of the *CCL2* gene. Summary of the SNPs located within HuR-binding regions is shown including SNP ID (rs#), genomic coordinates, strand, binding score, conservation scores (PhastCons, PhyloP), dataset accession numbers, alleles, and SNP position within peak region. Data was generated using POSTAR3 using the variation module. The GSE accession #s corresponds to those reported in the Gene Expression Omnibus (GEO).
