# Supplementary material for: The RNA-binding protein HuR modulates the expression of the disease-linked CCL2 rs1024611G-rs13900T haplotype
Source: eLife. 2026 Jan 14;13:RP93108. doi: 10.7554/eLife.93108 (PMC12803514; doi:10.7554/eLife.93108)
Supplement: Supplementary file 3. — A T:C ratio >1 indicates increased levels of the T allele relative to the C allele. [file elife-93108-supp3.docx]

|  | **gDNA** | **Cytosol** | **Monosome** | **Polysome** |
| --- | --- | --- | --- | --- |
| D-1 | 0.975806 | 0.872946 | 0.873425 | 1.778429 |
| D-2 | 1.034091 | 1.557778 | 1.441919 | 1.562622 |

**Supplementary File 3.** Loading of rs13900 alleles to cytosolic, monosomal and polysomal fractions from macrophage extracts prepared from heterozygous donors. T:C ratio > 1 indicates increased levels of the T allele relative to C allele.
